# Social Capital and Mental Health among Older Adults Living in Urban China in the Context of COVID-19 Pandemic

**DOI:** 10.3390/ijerph17217947

**Published:** 2020-10-29

**Authors:** Qian Sun, Nan Lu

**Affiliations:** 1 Department of Social Security, School of Public Administration, Hebei University of Economics and Business, Shijiazhuang 050061, China; sunqian@connect.hku.hk; 2 Sau Po Centre on Ageing, The University of Hong Kong, Hong Kong, China; 3 Department of Social Work and Social Policy, School of Sociology and Population Studies, Renmin University of China, Beijing 100872, China

**Keywords:** cognitive social capital, structural social capital, life satisfaction, depressive symptoms, older adults, urban China

## Abstract

Although social capital has been found to be an important social determinant of mental health in later life, research on social capital in the context of COVID-19 and the interplay among subdimensions of social capital is lacking. The present study examined the mediating role of cognitive social capital on the relationship between structural social capital and mental health among older adults in urban China in the context of the COVID-19 pandemic. Data were collected from the Yangpu district in Shanghai, China, in July–August 2020. A quota sampling approach was used to recruit 472 respondents aged 60 years and older from 23 communities in the Yangpu district. Mental health was measured by depressive symptoms and life satisfaction. Cognitive social capital was assessed through trust and reciprocity, and structural social capital was assessed through organization memberships, and COVID-19 related volunteering and citizenship activity. Structural equation modeling was used to test the mediation model. The results show that cognitive social capital had a full mediation effect on the association between structural social capital and mental health indicators (life satisfaction: *b* = 0.122, *SD* = 0.029, *p* < 0.001; depressive symptoms: *b* = −0.343, *SD* = 0.119, *p* < 0.01). The findings indicate that social capital can play an important role in sustaining and improving mental health in the context of the COVID-19 pandemic. Policy and intervention implications are discussed.

## 1. Introduction

The global pandemic of COVID-19 has had significant impacts on the health of the world’s older population [1,2,3]. Older adults are more vulnerable to COVID-19 than other age groups, mainly because of their relatively weak immune systems and chronic diseases. For example, the majority of COVID-19-related deaths occurred among older populations in both the United States and United Kingdom [1]. COVID-19 has become the second leading cause of mortality in the United States. Furthermore, the COVID-19 pandemic has significantly affected the mental health of older populations [1,2,4]. In particular, preventive strategies regarding COVID-19 often involve lockdowns of communities and social distancing, which could lead to social isolation, loneliness, and inadequate access to health resources [1,2,4,5]. In other words, older adults not only encounter a range of psychosocial stressors due to the COVID-19 pandemic (e.g., anxiety and uncertainty), but also have greater difficulty accessing social and medical resources and continuing their social participation in the communities [2,6]. Besides COVID-19, older adults are more likely to encounter other risk factors that threaten their mental well-being in later life, including but not limited to transitions in family roles, death of significant others, chronic diseases, and declining functional health [7,8]. It has become even more challenging to achieve healthy aging in the era of COVID-19. China has the largest population of adults aged 60 years or older in the world (253.88 million in 2019), According to the data from the China Mental Health Survey, the number of Chinese populations who suffer from depression was estimated to reach 95 million by 2015, around 16% of whom aged 65 years and older [9]. Therefore, research on the mental health of these Chinese older adults in the context of the COVID-19 pandemic could have important implications for future policy and intervention development concerning healthy aging, both nationally and internationally.

Mental health is recognized as a multidimensional concept which consists of two aspects: positive mental health and negative mental health [8]. Positive mental health refers to positive appraisal of well-being, supportive resources, and social networks, while negative mental health refers to mental illness. In this study, we use life satisfaction and depressive symptoms to measure positive mental health and negative mental health, respectively [8]. Both indicators not only are important criteria in evaluating healthy aging, but also play an important role in influencing older adults’ willingness to live in local communities and their capacities of fulfilling their social roles [10].

The literature has revealed social determinants of mental health in later life, including sociodemographic characteristics, financial status, living alone, family characteristics, and access to community services [11,12,13,14,15,16,17,18,19]. Furthermore, even as many older adults are instructed to self-quarantine and avoid face-to-face interactions with those who might infect them, their social connections in the community are one of the most important supportive resources during this difficult time. Community-based social capital (hereafter, social capital) is a modifiable and strong protective factor related to mental health among older populations [8,20,21,22,23]. Social capital is also considered an important component of COVID-19 strategies globally to reduce the social costs of individual actions and promote willingness to engage in collective actions to protect collective interests [4,6,24]. Although social capital is a culturally and contextually sensitive concept, its form in the context of COVID-19 is largely understudied. Many previous studies used a single indicator (e.g., social participation) to measure the multidimensional concept of social capital. Given the circumstances of China’s strict community-based prevention and control strategies related to the COVID-19 pandemic, social participation (which often requires frequent face-to-face interactions) might not be a suitable instrument to measure social capital. We argue that social capital can be viewed as a latent variable reflected by a range of observed indicators. Such an approach can provide a more comprehensive and accurate measurement of social capital. There is also a lack of research on the interplay between the subdimensions of social capital and mental health. Therefore, this study aimed to examine the latent construct of social capital in the context of COVID-19 and further test the relationship between multidimensional social capital and mental health among older adults in urban China.

### 1.1. The Theoretical Framework of Social Capital

There is a lack of consensus on the definition of social capital. Social capital can be viewed from an individual perspective and considered as a crucial social resource embedded in social connections in the community, through which people share memberships, value systems, and social norms [25,26]. Social capital can also be viewed as important community features, including but not limited to social trust, norms, and networks [27]. Social capital can be used to facilitate collective actions and pursue both individual and collective interests. In this study, social capital was assessed at an individual level, mainly due to variation in individuals’ access to community-based support resources such as services and amenities. Furthermore, the multidimensional construct of social capital can be divided into cognitive social capital and structural social capital [8]. Whereas the cognitive aspect refers to subjective self-evaluation of social connections such as social trust and perceived reciprocity among neighbors, the structural aspect is assessed by objective indicators such as involvement in social organizations, volunteering, social participation, and citizenship activities [8,28].

The COVID-19 pandemic not only brings great challenges in people’s daily lives, but also changes the way people interact with each other. Social capital could influence mental health among older adults during this difficult time through multiple mechanisms [8,20,21]: older adults could gain important social supportive resources through formal social organizations, collective activities, and informal reciprocity among neighbors. High social capital could also facilitate information diffusion in terms of home- and community-based services, which could further enhance older adults’ access to and use of these resources [29,30]. Thus, social capital is not only crucial for older adults to sustain and improve their mental health, but also can buffer adverse impacts of stressful events in older age. In addition, volunteering and citizenship activities could not only help older adults sustain their social network in the community, but also fulfil their social roles and enrich meaning in life [8,31]. In other words, social capital can prevent older adults from social withdraw and promote value, feelings of worthiness, and meaning in their daily lives, which are crucial for older adults’ mental health.

### 1.2. Cognitive Social Capital, Structural Social Capital, and Mental Health

The literature generally supports the important role of social capital in promoting mental health in later life [8,32]. However, findings regarding the associations between specific subdimensional social capital indicators and mental health have been inconclusive. Although some studies found that both cognitive social capital indicators (e.g., trust and reciprocity) and structural social capital indicators (e.g., organization membership and civic participation) are associated with life satisfaction and depressive symptoms [8,18,33,34,35,36], the associations between structural social capital and life satisfaction and depressive symptoms were found to be nonsignificant in European and East Asian contexts [8,17,20,32,37,38,39]. These inconsistencies might result from different conceptualizations, measurements, and analyses of social capital in previous studies. It might also be due to the lack of research on the interplay between cognitive and structural aspects of social capital and their effects on mental health.

The associations between cognitive and structural aspects of social capital have been identified in the literature [40,41,42,43]. These causal relationships could be bidirectional. Older adults with relatively high levels of social trust with their neighbors might be more likely to participate in social activities in local communities, and older adults who participate in volunteering and other community activities frequently might have more opportunities to foster social trust and reciprocity with their social connections in the community. We argue that the promotion of structural social capital could be a more practical approach to develop social capital in the community. Therefore, we aimed to not only test the direct effects of structural social capital and cognitive social capital on mental health in later life, but also went further by testing the role of cognitive social capital in the association between structural social capital and mental health.

Based on the literature and social capital theory, we hypothesized that cognitive social capital would play a mediating role in the relationship between structural social capital and mental health among older adults in China in the context of the COVID-19 pandemic.

## 2. Materials and Methods

### 2.1. Sampling

This research is based on the data from the Shanghai component of a community survey, Social Capital, Intergenerational Solidarity, and Mental Health among Older Chinese Adults (SCIENCE), which was jointly conducted by Renmin University and Hebei University of Economics and Business in 2020. The project of SCIENCE has been originally designed to collect data of social capital and healthy aging from urban and rural communities in China. After the COVID-19 pandemic outbreak, we modified the original data collection plan, added new COVID-19-related items in the questionnaire, and collected new data from Siping street, Yangpu district, Shanghai city from late July to early August 2020 (the levels of China’s street system are from communities to streets to districts). Shanghai is one of the most economically developed regions in China. Yangpu is in the northeastern part of the central area of Shanghai. Its GDP per capita ranked seventh among the city’s 16 districts in 2018. Siping street has 23 communities featuring around 105,000 residents, 34.17% of whom are aged 60 years or older. This figure is significantly higher than the national average (18.1% in 2019).

We applied a quota sampling approach to recruit respondents. First, all 23 communities from the Siping district were selected. Second, 20 respondents were recruited from each community based on referrals from community centers. The respondents needed to (1) be 60 years old or older, (2) have lived in the local community for more than 180 days in the past year, (3) have a local nonagricultural household registration status, and (4) had adequate cognitive capacities to complete the survey. The age and gender ratios of the sample were also controlled and consistent with a recent local representative sample collected by Shanghai Municipal Health Commission in 2019.

Face-to-face interviews were conducted in either community centers or the respondents’ homes. Informed consent forms were signed before the data collection. The survey collected rich information about sociodemographic characteristics, mental health, physical diseases, functional health, family characteristics, and social capital. The response rate was higher than 90% in all communities. The Short Portable Mental Status Questionnaire was used to determine whether the respondents had adequate cognitive function to complete the survey [44]. Ethics approval was obtained from the Ethics Committee of the University of Hong Kong (EA200113). Research data are available in the Supplementary Materials (File S1).

### 2.2. Measurements

#### 2.2.1. Outcome Variable

We used the Life Satisfaction Scale for Chinese older adults to assess life satisfaction [45]. The scale has eight items asking respondents to assess their satisfaction levels in eight important life domains: family interactions, family ties, relationship with spouse, friendship, housing, health, food, and financial status. Responses were assessed on a 5-point Likert scale ranging from 1 (strongly disagree) to 3 (fair) to 5 (strongly agree). Mean scores were calculated to represent the level of life satisfaction (Cronbach’s alpha = 0.837).

Depressive symptoms include a range of symptoms such as depressive moods, loss of interest in social activities that she or he used to enjoy, feelings of fatigue, changes in appetite and sleeping, difficulty in concentrating and thinking, and even suicidal thoughts. In this study, depressive symptoms were measured by the 10-item Center on Epidemiologic Studies Depression Scale [46]. In specific, the respondents were asked whether they were bothered by things that generally did not bother them, had difficulty in keeping their minds on daily tasks, felt everything was an effort, and even could not get “going” and fulfill their social roles in daily lives. They were also asked whether they felt fearful, depressed, lonely, happy, and hopeful about their future, and had poor quality of sleep. The responses were measured by a five-point Likert scale (0 = less than 1 day, 2 = 3 to 4 days, and 4 = almost every day). The summed scores were calculated to represent the levels of depressive symptoms (Cronbach’s alpha = 0.726).

#### 2.2.2. Social Capital Variable

Cognitive and structural social capital were treated as latent variables, meaning that these two constructs cannot be directly observed but can be assessed using other observed indicators [47]. Social capital indicators were selected and modified based on the short Social Capital Assessment Tool and an integrated questionnaire for social capital from the World Bank [28,48]. The above measurement tools have been widely applied in Chinese social capital studies and are recommended to be used in the contexts of low- and middle-income countries [37,38,49]. The latent variable of cognitive social capital was assessed through four indicators. Respondents were asked whether they agreed with the following statements based on their life experiences in local communities during the COVID-19 pandemic outbreak (from 23 January to 8 June 2020; local community centers were reopened and community activities resumed on June 8 in Shanghai): (1) they trust the majority of local residents in the community; (2) they considered the local community as a big family and had a sense of belonging to the community; (3) local residents helped each other when necessary; and (4) local residents care about both their own benefits and others’ interests. The responses were assessed on a 5-point Likert scale, ranging from 1 (strongly disagree) to 3 (neutral) to 5 (strongly agree).

In this study, the latent variable of structural social capital was assessed through the following three indicators: number of organization memberships, citizenship activities, and volunteering activities during the COVID-19 pandemic outbreak [28,48]. Specifically, respondents were asked whether they held memberships in the following organizations: political parties (Communist Party of China, and the Democratic Parties), labor unions, universities for older adults, neighborhood committees, community associations, women’s groups, charity groups, religious groups, sports groups, and professional associations. The answers were assessed by binary variables (0 = no, 1 = yes) and scores were summed, ranging from 0 to 11. Furthermore, respondents were asked whether they worked with other local residents to cope with common problems during the pandemic outbreak (1 = never participated, 3 = occasionally, 5 = participated in almost all activities). Finally, they were asked whether they engaged in any formal volunteering activity during this period, including providing support to vulnerable groups (older adults, children, and disabled people who need support); COVID-19-related volunteering activities (e.g., temperature check, education programs regarding COVID-19 prevention and control); and other types of volunteering activities that served the interests of the local community. Responses to each volunteering activity type were assessed as binary variables (0 = no, 1 = yes). Summed scores were calculated to represent the level of volunteering participation.

#### 2.2.3. Covariates

Age was assessed by subtracting the respondents’ birth year from the survey year. Gender, marital status, and educational attainment were assessed by binary variables (0 = men, 1 = women; 0 = other marital status, 1 = married; 0 = secondary school education or lower, 1 = high school education or higher). Number of children was assessed by the number of sons and daughters. Log values of the respondents’ monthly household income were calculated. Furthermore, we used the Lawton Instrumental Activities of Daily Living to measure instrumental activities of daily living [50]. The respondents were asked to report on 13 types of doctor-diagnosed chronic diseases, such as cardiovascular diseases, stomach or other digestive diseases, and hypertension. The responses were assessed by binary variables (0 = no, 1 = yes). We summed the scores to measure the number of chronic diseases. This method has been used in previous research [51].

### 2.3. Statistical Analysis

Structural equation modeling was used to test the mediating role of cognitive social capital in the relationship between structural social capital and mental health. A two-step analytic procedure was applied. The first step was to establish the measurement model of cognitive social capital and structural social capital in an urban Chinese context during the COVID-19 pandemic outbreak. We used the following fit indexes to assess the model fit: chi-square test statistic, root mean square error of approximation (RMSEA), Tucker–Lewis index (TLI), comparative fit index (CFI), and standardized root mean square residual (SRMR) [47]. We used the following criteria to test the model fit: nonsignificant chi-square estimate, TLI and CFI > 0.95, and RMSEA and SRMR < 0.05 [47,52].

In the second step, we conducted two structural models. In the first structural model, life satisfaction and depressive symptoms were regressed on structural social capital, while controlling for covariates. Cognitive social capital was added to the second model. In doing so, we tested the indirect effect of structural social capital on life satisfaction and depressive symptoms through cognitive social capital. Mplus 7.0 (Muthén and Muthén, Los Angeles, CA, USA) was used to assist the data analysis [53].

## 3. Results

### 3.1. Descriptive Statistics

The sample characteristics are illustrated in Table 1. The average age of the respondents was 68.37 years. More half of the respondents were women, 83.1% were married, 57.8% completed a high school education or higher, and 12.7% lived alone. Monthly household income was higher than 5000 RMB for 83.0% of participants. On average, each respondent had 1.18 adult children; 76.7% had one adult child. Respondents had 1.36 chronic diseases on average; 29.7% did not have any chronic diseases at the time of the survey. Most respondents (93.2%) did not have limitations in their instrumental activities of daily living. Furthermore, approximately 24.6% of the respondents did not report any depressive symptoms. On average, 74.8% of the respondents were satisfied or very satisfied with their lives.

In general, the majority of older residents reported high levels of trust and reciprocity in the community. For example, around 90% of the respondents agreed or strongly agreed that the majority of local residents can be trusted, cared about other people’s interests, and helped each other out, and considered local communities as big families. Furthermore, 28.4% of the respondents have participated in social volunteering activities related to COVID-19 during the COVID-19 pandemic. Of the respondents, 35.6% reported that they had collaborated with other residents to solve common problems in the communities during this difficult time. On average, each respondent had 2.58 organization memberships.

### 3.2. Structural Equation Modeling

The measurement model of cognitive and structural social capital was established before we conducted the structural model. Fit index estimates suggested a good model fit: χ^2^(10) = 8.968, *p* = 0.5352, RMSEA = 0.000, CFI = 1.000, TLI = 1.001, SRMR = 0.008. Furthermore, the standardized estimates of the factor loadings ranged from 0.869 to 0.913 for cognitive social capita, and from 0.586 to 0.702 for structural social capital. We present the measurement model in Table 2.

In the second step, we conducted the first structural model by regressing life satisfaction and depressive symptoms on structural social capital. The estimates of fit indexes indicated that the model adequately fit the data: χ^2^(3) = 1.072, *p* = 0.7840, RMSEA = 0.000, CFI = 1.000, TLI = 1.011, SRMR = 0.006. Structural social capital was significantly associated with life satisfaction and depressive symptoms (life satisfaction: *b* = 0.138, *SD* = 0.043, *p* < 0.01; depressive symptoms: *b* = −0.524, *SD* = 0.266, *p* < 0.05).

**Figure 1:**
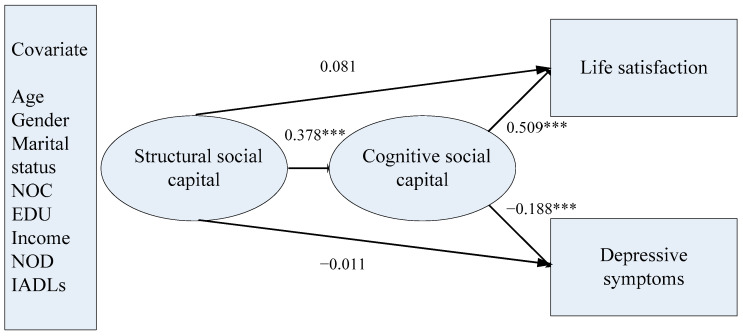
The structural model of social capital and mental health. *Notes:* Only standardized coefficients are reported. *** *p* < 0.001 (two-tailed); NOC = number of children; EDU = educational attainment; NOD = number of chronic diseases; IADL = instrumental activities of daily living.

In the second structural model, life satisfaction and depressive symptoms was regressed on the cognitive social capital and structural model, controlling for covariates (χ^2^(61) = 76.146, *p* = 0.0916, RMSEA = 0.023, CFI = 0.994, TLI = 0.990, SRMR = 0.020). Structural social capital was no longer a significant factor for life satisfaction and depressive symptoms (life satisfaction: *b* = 0.051, *SD* = 0.037, *p* = 0.164; depressive symptoms: *b* = −0.055, *SD* = 0.319, *p* = 0.864). Cognitive social capital was significantly associated with life satisfaction and depressive symptoms (life satisfaction: *b* = 0.447, *SD* = 0.040, *p* < 0.001; depressive symptoms: *b* = −1.251, *SD* = 0.341, *p* < 0.001). Furthermore, structural social capital was significantly associated with cognitive social capital (*b* = 0.274, *SD* = 0.060, *p* < 0.001). The mediation effects of cognitive social capital on the relationships between structural social capital and life satisfaction and depressive symptoms were both statistically significant (life satisfaction: *b* = 0.122, *SD* = 0.029, *p* < 0.001; depressive symptoms: *b* = −0.343, *SD* = 0.119, *p* < 0.01). We present the final model in Figure 1.

## 4. Discussion

Older adults are vulnerable during the COVID-19 pandemic and at higher risk of poor mental health. Social capital from communities could play an even more important role in sustaining the mental health of older adults in this difficult period. This study added new empirical evidence to support the proposed mediation model from the perspective of social capital theory.

We established two comprehensive latent constructs of social capital based on data recently collected in Shanghai. By definition, the findings show that high cognitive social capital was associated with high levels of trust and reciprocity, whereas high structural social capital was reflected by memberships in organizations and frequent participation in COVID-19-related volunteering and citizenship activities [28,48]. Whereas the literature features mixed findings on the association between structural social capital and mental health [8,20,32,33,35,37,38,39], the findings of this study represent new contributions by demonstrating the mediating effect of structural social capital on life satisfaction and depressive symptoms through fostering cognitive social capital. This could be because high levels of structural social capital mean more opportunities for older adults to enhance their trust and perceived reciprocity with other residents. High levels of trust and reciprocity not only reflect good-quality social relationships, but also promote meaning in life and positive thoughts. These factors are important for improving life satisfaction levels and reducing depressive symptoms among older adults.

These findings have policy and intervention implications. First, social capital latent constructs should be included in screening tools to identify community-dwelling older adults at risk of poor mental health in the context of the COVID-19 pandemic. Second, cognitive social capital can be promoted through fostering trust and reciprocity among residents in the community. Enhancing community organizations and promoting older adults’ participation in community-based social volunteering and citizenship activities (which were related to COVID-19 such as temperature check, provision of support to vulnerable groups, and educational programs in terms of preventing and controlling COVID-19) are an effective approach to promoting structural social capital. Older residents, especially for the young-old groups, should be encouraged to participate in such activities if these activities do not increase the risk of older adults’ exposure to COVID-19. Such a strategy is also found to be an effective method to solve the shortage of human resources in the community during the COVID-19 pandemic. Third, future social capital policies and interventions should emphasize cognitive social capital. In other words, the promotion of structural social capital should lead to enhanced trust and reciprocity. For example, participation in COVID-19-related volunteering activities could not only promote older adults’ feelings of belongingness in the community, trust with other residents, and meaning in life, but also help them utilize all available resources to provide support for people in need during this difficult time (e.g., young–old adults could provide support to old–old adults).

The present study has the following limitations. First, the data were cross-sectional in nature. Therefore, causality in the relationship between cognitive social capital and structural social capital cannot be tested in this study. However, theoretical discussions were provided for the proposed mediation model. Longitudinal studies with larger sample sizes are needed to further examine the interplay among cognitive social capital, structural social capital, and mental health, as well as their trajectory patterns. Furthermore, we cannot examine the effects of the COVID-19 pandemic on life satisfaction and depressive symptoms among older respondents living in urban Chinese communities. The prevalence rates of depression and depressive symptoms and the levels of life satisfaction among older populations vary across Chinese empirical studies, mainly because of the inconsistency in the measurement instruments and differences in sample characteristics (e.g., age and region) [54]. Therefore, it is difficult to determine the changes in depressive symptoms and life satisfaction in Shanghai before and after the COVID-19 pandemic outbreak period. Future longitudinal studies are needed to examine the effect of the COVID-19 pandemic on mental health among older adults. It is also important to develop interventions to sustain or improve mental health among older adults who are severely affected by the COVID-19 pandemic. The present study is one of the first attempts to examine the role of social capital in influencing mental health in later life in the context of the COVID-19 pandemic. The findings suggest that social capital can play an important role in both prevention and intervention programs concerning mental health in older age. Moreover, we did not use random sampling strategies to recruit respondents in communities in Shanghai, which limits the empirical generalization of the study’s findings. Shanghai is one of the most economically developed cities in China. The level of population aging in Shanghai is also one of the highest in China. Therefore, the empirical generalization of the findings should be confined to those with similar demographic, social and economic backgrounds. Future studies are needed to examine social capital and mental health in later life in other social contexts (e.g., rural areas and small cities). Furthermore, all key variables were self-reported, which could affect the level of information accuracy and lead to misclassification bias. Finally, social capital was assessed at an individual level in this research. Future studies are needed to examine social capital from a collective level (e.g., community and nation) in the COVID-19 context.

## 5. Conclusions

The present study tested the mediation role of cognitive social capital in the association between structural social capital and mental health among older adults in urban China. The latent variables of cognitive social capital and structural social capital were established using observed variables that were culturally and contextually sensitive to urban Chinese contexts in the context of the COVID-19 pandemic. The findings indicate that the association between structural social capital and mental health in later life was statistically significant in the context of the COVID-19 pandemic. Cognitive social capital played a full mediation role in this association. Social capital latent variables should be considered as an important screening tool and included in community-based prevention and control efforts related to the COVID-19 pandemic. The future development of social capital policies and interventions should emphasize the interplay between structural social capital and cognitive social capital, as well as their influence on mental health.

## Figures and Tables

**Table 1:** Sample Characteristics (*N* = 472).

	*N* (%)	*M* (*SD*)
Age		68.37 (6.69)
60–70	329 (69.7)	
71 or older	142 (30.1)	
Gender		
Men	206 (43.6)	
Women	266 (56.4)	
Married	392(83.1)	
Educational attainment		
Secondary school or lower	199 (42.2)	
High school or above	273 (57.8)	
Monthly household income		
0–9999 RMB	224 (47.5)	
10,000 RMB or more	235 (49.8)	
Number of children		1.19 (0.62)
Number of chronic diseases		1.37 (1.41)
IADL		0.02 (0.10)
Life satisfaction		4.27 (0.48)
Depressive symptoms		3.14 (3.64)

Notes: 100 RMB = $14.54 USD; IADL = instrumental activities of daily living.

**Table 2:** Measurement Model of Cognitive Social Capital and Structural Social Capital.

	Estimate	*SD*	Standardized Estimate	*SD*
Cognitive social capital				
Trust in local community	1.000	0.000	0.869 *	0.040
Perceived helpfulness of others	0.995 *	0.095	0.897 *	0.042
Willingness to cooperate with others	1.021 *	0.097	0.913 *	0.042
Feelings of belonging	0.874 *	0.029	0.884 *	0.042
Structural social capital				
Organization memberships	1.000	0.000	0.586 *	0.073
Citizenship activities	1.246 *	0.297	0.625 *	0.081
Volunteering	0.662 *	0.153	0.702 *	0.083

* p < 0.001 (two-tailed).

## Supplementary Materials

The following are available online at https://www.mdpi.com/1660-4601/17/21/7947/s1, File S1: Research Data.

## References

[1] Wu B. (2020). Social isolation and loneliness among older adults in the context of COVID-19: A global challenge. Glob. Health Res. Policy.

[2] Marroquín B., Vine V., Morgan R. (2020). Mental health during the COVID-19 pandemic: Effects of stay-at-home policies, social distancing behavior, and social resources. Psychiatry Res..

[3] Rajkumar R.P. (2020). COVID-19 and mental health: A review of the existing literature. Asian J. Psychiatry.

[4] Rodela T.T., Sultana A., McKyer E.L.J., Bhattacharya S., Hossain M. Social Capital and Mental Health during the COVID-19 Pandemic. osf.io/preprints/socarxiv/zrkgh.

[5] World Health Organization. Global Recommendations on Physical Activity for Health. https://www.who.int/dietphysicalactivity/factsheet_recommendations/en/.

[6] Bian Y., Miao X., Lu X., Ma X., Guo X. (2020). The Emergence of a COVID-19 Related Social Capital: The Case of China. Int. J. Sociol..

[7] Kim D., Baum C.F., Ganz M., Subramanian S., Kawachi I. (2011). The contextual effects of social capital on health: A cross-national instrumental variable analysis. Soc. Sci. Med..

[8] Nyqvist F., Forsman A.K., Giuntoli G., Cattan M. (2012). Social capital as a resource for mental well-being in older people: A systematic review. Aging Ment. Health.

[9] Huang Y., Wang Y., Wang H., Liu Z., Yu X., Yan J., Yu Y., Kou C., Xu X., Lu J. (2019). Prevalence of mental disorders in China: A cross-sectional epidemiological study. Lancet Psychiatry.

[10] Lou V.W.Q. (2015). Spiritual Well-Being of Chinese Older Adults Conceptualization, Measurement and Intervention.

[11] Kim H.J., Hong S., Kim M. (2015). Living arrangement, social connectedness, and life satisfaction among Korean older adults with physical disabilities: The results from the national survey on persons with disabilities. J. Dev. Phys. Disabil..

[12] Li C., Chi I., Zhang X., Cheng Z., Zhang L., Chen G. (2015). Urban and rural factors associated with life satisfaction among older Chinese adults. Aging Ment. Health.

[13] Li H., Chi I., Xu L. (2013). Life satisfaction of older Chinese adults living in rural communities. J. Cross-Cult. Gerontol..

[14] Ng S.T., Tey N.P., Asadullah M.N. (2017). What matters for life satisfaction among the oldest-old? Evidence from China. PLoS ONE.

[15] Lu N., Spencer M., Sun Q., Lou V.W.Q. (2020). Family social capital and life satisfaction among older adults living alone in urban China: The moderating role of functional health. Aging Ment. Health.

[16] Lu N., Xu L., Lou V.W.Q., Chi I. (2018). Intergenerational relationships and the trajectory of depressive symptoms among older Chinese adults in rural migrant families. Aging Ment. Health.

[17] Bassett E., Moore S. (2013). Social capital and depressive symptoms: The association of psychosocial and network dimensions of social capital with depressive symptoms in Montreal, Canada. Soc. Sci. Med..

[18] Cao W., Li L., Zhou X., Zhou C. (2015). Social capital and depression: Evidence from urban elderly in China. Aging Ment. Health.

[19] Schwarzbach M., Luppa M., Forstmeier S., Konig H., Riedelheller S.G. (2014). Social relations and depression in late life-A systematic review. Int. J. Geriatr. Psychiatry.

[20] Lu N., Jiang N., Lou V.W.Q., Zeng Y., Liu M. (2018). Does gender moderate the relationship between social capital and life satisfaction? Evidence from urban China. Res. Aging.

[21] Lu N., Lum T.Y.S., Lou V.W.Q. (2016). The impacts of community social capital on the life satisfaction of older adults in Hong Kong: The moderator role of intergenerational family capital. Aging Ment. Health.

[22] Lu N., Peng C. (2019). Community-based structural social capital and depressive symptoms of older urban Chinese adults: The mediating role of cognitive social capital. Arch. Gerontol. Geriatr..

[23] Zhang J.Y., Lu N. (2019). Community-based cognitive social capital and depressive symptoms among older adults in urban China: The moderating role of family social capital. Int. J. Aging Hum. Dev..

[24] Bartscher A.K., Seitz S., Sieglich S., Slotwinski M., Wehrhöfer N., Siegloch S. Social Capital and the Spread of Covid-19: Insights from European Countries. https://ideas.repec.org/p/ces/ceswps/_8346.html.

[25] Coleman J.S. (1988). Social capital in the creation of human capital. Am. J. Sociol..

[26] Bourdieu P., Richardson J.G. (1986). The Forms of Capital. Handbook of Theory and Research for the Sociology of Education.

[27] Putnam R.D., Leonardi R., Nanetti R. (1993). Making Democracy Work: Civic Traditions in Modern Italy.

[28] De Silva M.J., Huttly S.R., Harpham T., Kenward M.G. (2007). Social capital and mental health: A comparative analysis of four low income countries. Soc. Sci. Med..

[29] Sun Q., Lu N., Jiang N., Lou V.W.Q. (2020). Intention to use respite services among informal care-givers of frail older adults in China: The role of care needs change. Ageing Soc..

[30] Peng C., Burr J.A., Kim K., Lu N. (2020). Home and community-based service utilization among older adults in urban China:The role of social capital. J. Gerontol. Soc. Work.

[31] Lu N., Jiang N., Sun Q., Lou V.W.Q. (2019). Community social capital and positive caregiving experiences among adult-children caregivers of older adults with disabilities in urban China. Res. Aging.

[32] Ehsan A., Klaas H.S., Bastianen A., Spini D. (2019). Social capital and health: A systematic review of systematic reviews. Ssm-Popul. Health.

[33] Ajrouch K. (2007). Resources and well-being among Arab-American elders. J. Cross-Cult. Gerontol..

[34] Theurer K., Wister A. (2010). Altruistic behaviour and social capital as predictors of well-being among older Canadians. Ageing Soc..

[35] Yamaoka K. (2008). Social capital and health and well-being in East Asia: A population-based study. Soc. Sci. Med..

[36] Chiao C., Weng L., Botticello A.L. (2011). Social participation reduces depressive symptoms among older adults: An 18-year longitudinal analysis in Taiwan. BMC Public Health.

[37] Yip W., Subramanian S.V., Mitchell A.D., Lee D.T.S., Wang J., Kawachi I. (2007). Does social capital enhance health and well-being? Evidence from rural China. Soc. Sci. Med..

[38] Norstrand J.A., Xu Q. (2011). Social capital and health outcomes among older adults in China: The urban–rural dimension. Gerontologist.

[39] De Silva M.J., McKenzie K., Harpham T., Huttly S. (2005). Social capital and mental illness: A systematic review. J. Epidemiol. Community Health.

[40] Brehm J., Rahn W.M. (1997). Individual-level evidence for the causes and consequences of social capital. Am. J. Political Sci..

[41] Lindstrom M. (2004). Social capital, the miniaturisation of community and self-reported global and psychological health. Soc. Sci. Med..

[42] Lu N., Peng C., Jiang N., Lou V.W.Q. (2018). Cognitive social capital and formal volunteering among older adults in urban China: Does gender matter?. J. Appl. Gerontol..

[43] Lu N., Zhang J. (2019). Social capital and self-rated health among older adults living in urban China: A mediation model. Sustainability.

[44] Pfeiffer E. (1975). A Short Portable Mental Status Questionnaire for the assessment of organic brain deficit in elderly patients. J. Am. Geriatr. Soc..

[45] Lou V.W.Q., Chi I., Mjelde-Mossey L.A. (2008). Development and validation of a life satisfaction scale for Chinese elders. Int. J. Aging Hum. Dev..

[46] Rankin S.H., Galbraith M.E. (1993). Reliability and validity data for a Chinese translation of the Center for Epidemiological Studies-Depression. Psychol. Rep..

[47] Kline R.B. (2011). Principles and Practice of Structural Equation Modeling.

[48] Grootaert C., Narayan D., Jones V.N., Woolcock M. (2004). Integrated Questionnaire for the Measurement of Social Capital.

[49] Agampodi T.C., Agampodi S.B., Glozier N., Siribaddana S. (2015). Measurement of social capital in relation to health in low and middle income countries: A systematic review. Soc. Sci. Med..

[50] Lawton M.P., Brody E.M. (1969). Assessment of older people: Self-maintaining and instrumental activities of daily living. Gerontologist.

[51] Lu N., Wu B., Jiang N., Dong T. (2020). Association between childhood conditions and arthritis among middle-aged and older adults in China: The China Health and Retirement Longitudinal Study. Ageing Soc..

[52] Bentler P.M. (1990). Comparative fit indexes in structural models. Psychol. Bull..

[53] Muthén L.K., Muthén B. (2012). Mplus User’s Guide.

[54] Qin X., Wang S., Hsieh C. (2016). The prevalence of depression and depressive symptoms among adults in China: Estimation based on a National Household Survey. China Econ. Rev..

